# Management of Postoperative Myocardial Injury After Non-cardiac Surgery in Patients Aged ≥ 80 Years: Our 10 Years' Experience

**DOI:** 10.3389/fcvm.2022.869243

**Published:** 2022-04-13

**Authors:** Linggen Gao, Lei Chen, Bin Wang, Jing He, Chaoyang Liu, Rong Wang, Rui Cheng

**Affiliations:** ^1^Department of Comprehensive Surgery, General Hospital of Chinese People's Liberation Army and National Clinical Research Center for Geriatric Disease, Beijing, China; ^2^Department of Thoracic Surgery, General Hospital of Chinese People's Liberation Army, Beijing, China

**Keywords:** postoperative myocardial injury, non-cardiac surgery, comanagement care model, management strategy, prognosis

## Abstract

**Background:**

Postoperative myocardial injury (PMI) is associated with short- and long-term mortality. The incidence of PMI in very old patients is currently unknown. There is currently neither known effective prophylaxis nor a uniform strategy for the elderly with PMI.

**Objective:**

To share our 10 years of experience in the comprehensive management of PMI after non-cardiac surgery in patients aged ≥ 80 years.

**Methods:**

In this case series, we retrospectively collected and assessed the 2,984 cases aged ≥ 80 years who accepted non-cardiac surgery from 2011 to 2021 at the second Medical Center, Chinese PLA General Hospital. The incidence, risk factors, management strategy, and prognosis of surgical patients with PMI were analyzed.

**Results:**

A total of 2,984 patients met our inclusion criteria. The overall incidence of PMI was 14%. In multivariable analysis, coronary artery disease, chronic heart failure, and hypotension were independently associated with the development of PMI. The patients with PMI were at a higher risk of death (OR, 2.69; 95% CI, 1.78–3.65). They were more likely to have received low molecular heparin, anti-plantlet therapy, beta-blocker, early coronary angiography, and statin than patients without PMI. The 30-day (0.96% vs. 0.35%; OR 3.46; 95% CI, 1.49–7.98; *P* < 0.001) and 1-year mortality (5.37% vs. 2.60%; OR 2.35; 95% CI, 1.12–6.53; *P* < 0.001) was significantly higher in patients with PMI compared with those without PMI.

**Conclusions:**

The incidence of PMI in very old patients was high. The PMI is associated with an increased risk of 30 days and 1-year mortality. These patients can benefit from intensification of assessment and individualized care of multi-morbidities during the perioperative period. Especially cardiovascular medical treatments, such as antiplatelet, anticoagulation, β-blockers, and statins are very important for patients with PMI.

## Introduction

As the population ages, the rate of surgical procedures in the older population is rising. Aged patients, especially those over 80 years, are more likely to suffer from comorbidities, such as coronary heart disease, hypertension, diabetes, polypharmacy, and decline of organ function. Thus, to ensure surgical safety, the perioperative management team is of great significance. The department of geriatric comprehensive surgery of the author's team is composed of geriatric physicians, who have been engaged in geriatric perioperative management for 35 years. We have established a co-management care model by geriatricians and surgeons, ensuring the perioperative safety of geriatric surgical patients and accumulating rich clinical experience.

Considering the advancing age and co-morbid status of the surgical patient cohort, the incidence of postoperative cardiac complications will likely continue to rise in the aging population. Postoperative myocardial injury (PMI) after non-cardiac surgery continues to pose challenges to geriatric physicians with regards to a comprehensive diagnostic and management approach. According to the VISION study, so far, the largest prospective study performed in non-cardiac surgery (1), PMI has been identified as troponin elevation within 30 days after non-cardiac surgery, which is associated with an increased risk of mortality and major vascular complications at 30 days and up to 2 years after non-cardiac surgery (1–5). Recent studies have shown that more than 85% of PMIs are missed in clinical practice when relying on the reporting of typical symptoms. Therefore, PMIs can only be reliably detected with perioperative screening using sensitive or high-sensitivity cardiac troponin T/I measurements (6). The estimated incidence of myocardial injury after non-cardiac surgery based on data from 44 high quality and prospective studies, which routinely measured troponin after surgery, is 19.5% (7). However, the incidence of PMI in old patients is currently unknown. At present, there are few reports on myocardial injury after non-cardiac surgery in people over 80 years old and there is still a lack of treatment strategy for PMI. We, therefore, aimed to investigate the incidence of PMI in old patients, as well 30-day and 365-day mortality, and to summarize the clinical experience of the department for 10 years, to provide a reference for the prevention and treatment of PMI.

## Methods

### Study Population

Patients in this study were drawn from the Second Medical Center, Chinese PLA General Hospital. We included consecutive patients undergoing non-cardiac surgery who were at least 80 years of age and had a troponin measurement within the first 24 h and 3 days after surgery between January 1, 2011, and January 1, 2021.

### Data Collection

Data were collected from electronic health records (EHR) from our hospital. The variables collected included demographics, laboratory measurements, troponin I concentrations, disease diagnosis, comorbidities, procedures, and outcomes. Comorbidities were extracted using International Classification of Disease-Revision 9/10 billing codes for atrial fibrillation (AF), asthma, coronary artery disease (CAD), cancer, chronic kidney disease (CKD), chronic obstructive pulmonary, diabetes (DM), heart failure (HF), and hypertension (HTN). Troponin T concentrations were assessed and the reference level for normal is <0.03 ng/ml. The clinical follow-up data were obtained using direct contact with the patients, their referring physicians, and family physicians.

### Evaluation of PMI

The PMI was defined as a peak plasma troponin T concentration of 0.03 ng/ml or greater than resulted from myocardial ischemia within 3 days after surgery. The pre-operative assessment was carried out by physicians.

### Individualizing Risk Assessment and Care in Patients With Associated Conditions and Comorbidities

Trained research personnel reviewed the charts to obtain information on potential preoperative predictors of major perioperative complications by using standardized definitions. The Revised Cardiac Risk Index (RCRI) was calculated in all patients to estimate cardiovascular risk. The RCRI has six predictors of risk as to the following: creatinine ≥2 mg/dl, heart failure, insulin-dependent diabetes mellitus, intrathoracic, intra-abdominal, or suprainguinal vascular surgery, history of cerebrovascular accident or Transient Ischaemic Attack (TIA), and ischemic heart disease. Each predictor is worth 1 point. Patients with more predictors of risk would have a higher risk.

Postoperative pulmonary complications were evaluated by using a seven-variable risk assessment tool Assess Respiratory Risk in Surgical Patients in Catalonia (ARISCAT). The ARISCAT score included seven independent risk factors: low preoperative arterial oxygen saturation, acute respiratory infection during the previous month, age, preoperative anemia, upper abdominal or intrathoracic surgery, surgical duration of at least 2 h, and emergency surgery (8).

Hypotension was defined as a mean arterial pressure (MAP) below 65 mm Hg for at least 1 min (9).

Major bleeding was defined using modified International Society on Thrombosis and Haemostasis (ISTH) criteria to include any one of the following: (1) fatal bleeding, (2) bleeding into a critical area or organ, (3) fall in hemoglobin of ≥ 2 g/dl over 24 hours, (4) transfusion of two or more units of whole blood or packed red blood cells over 24 h, and in surgical patients, and (5) surgical site bleeding that required a second intervention (10).

We excluded patients who were incorrectly screened (<80 years, <24-h hospital stays), had their surgery canceled, had cardiac surgery or MI within 14 days before surgery. For the analysis addressing 30-day and 1-year mortality, we only included every patient once at first enrollment.

### Statistical Analysis

Descriptive analyses were performed by troponin T levels stratified into normal and elevated groups (more than the upper limit of normal, >0.03 ng/ml). We used troponin measurements within 3 days after the operation. If multiple troponin measurements were available within 24 h, the patient's highest measurement was used. Categorical variables were reported as total count and percentage of patients. Continuous no troponin laboratory values were reported as median and interquartile range. The characteristics of patients who did and did not develop PMI were compared using the Chi-square test for categorical variables and the Student's *t*-test for continuous variables. Multivariable logistic regression analysis was used to estimate the independent predictors of myocardial injury after non-cardiac surgery. We report the 30-day mortality and other important 30-day outcomes (non-fatal cardiac arrest, congestive heart failure, stroke, and composite of major events) for patients with and without PMI. To assess the effects of PMI on outcomes, we conducted a survival analysis with the dependent variable of time to mortality, setting the time of days after the operation.

## Results

### Study Cohort and Patient Characteristics

A total of 2,984 surgeries met our inclusion criteria. The baseline characteristics of the surgical patients who did and did not develop PMI are reported in Table 1. All the patients underwent troponin measurement every day within 3 days after the operation. Approximately 50% of PMI occurs on the day of surgery, and 18% on the second postoperative day. The mean [±standard deviation (SD)] age of the patients was 83.7 ± 8.25 years. The majority of participants were classified as either ASA class 2 (70.38%) or ASA class 3 (23.39%) before surgery. More often, patients with PMI had coronary artery disease, atrial fibrillation, peripheral artery disease, prior stroke, diabetes mellitus, chronic obstructive pulmonary disease (COPD), active tumor disease, and had a higher ARISCAT score than patients who did not develop PMI.

**Table 1:** Baseline characteristics.

	**All Patients (*n* = 2,984)**	**PMI (*n* = 418)**	**No PMI (*n* = 2,566)**	***P*-Value**
Age	83.7 ± 8.25	83.5 ± 8.15	83.9 ± 8.30	0.371
Sex, male	2,730 (91)	356 (85.17)	1,143 (89.09)	0.102
ASA				
I	118 (3.95)	10 (2.39)	108 (4.21)	
II	2,100 (70.38)	256 (6.12)	1,844 (71.86)	
III	698 (23.39)	76 (18.18)	622 (24.24)	
IV	68 (2.28)	14 (3.34)	54 (2.10)	0.423
≥4 MET	1,910 (64.0)	272 (65.07)	1,638 (63.83)	0.730
Clinical covariates				
Body mass index, kg/m^2^	26.4 ± 2.5	26.9 ± 2.9	25.8 ± 2.1	0.745
ACE inhibitor or ARB use	1,064 (35.66)	168 (40.19)	896 (34.92)	0.140
Statin use	1,478 (49.53)	216 (51.67)	1,182 (46.06)	0.000
Comorbidities				
Coronary artery Disease	972 (32.57)	310 (74.16)	662 (25.80)	0.000
Prior myocardial Infarction	394 (13.20)	178 (42.58)	216 (8.42)	0.000
Chronic heart failure	828 (27.75)	206 (49.28)	622 (24.24)	0.000
Chronic kidney disease	858 (28.75)	140 (33.49)	718 (27.98)	0.103
Atrial fibrillation	326 (10.92)	166 (39.71)	160 (6.23)	0.000
Peripheral artery Disease	1,182 (39.61)	372 (88.99)	810 (31.57)	0.000
Hypertension	1,534 (51.41)	216 (51.67)	1,318 (51.36)	0.934
Prior stroke/TIA	1,304 (43.70)	218 (52.15)	1,086 (42.32)	0.008
Diabetes mellitus	1,268 (42.50)	226 (54.07)	1,042 (40.60)	0.000
COPD	1,058 (35.46)	236 (56.46)	822 (32.03)	0.000
Asthma	254 (8.51)	36 (8.61)	218 (8.50)	0.955
ARISCACT score	28 ± 8	32 ± 5	26 ± 7	0.035
Active tumor disease	1,274 (42.69)	108 (25.84)	1,166 (45.44)	0.000
RCRI class				
I	210 (7.04)	40 (9.57)	170 (6.62)	
II	1,792 (60.05)	308 (73.68)	1,484 (57.83)	
III	727 (24.40)	60 (14.35)	668 (26.03)	
IV	74 (2.48)	10 (2.39)	64 (2.49)	
V	1 (0)	0	0	0.000
Elective surgery	2,506 (77.28)	246 (58.85)	2,060 (80.28)	0.000
Emergency surgery, ≤ 24 h	428 (14.34)	116 (27.75)	312 (12.16)	0.000
Urgent surgery, >24 h	250 (8.38)	56 (13.40)	194 (7.56)	0.316
Type of Surgery				
General Surgery	542 (18.16)	125 (29.9)	417 (16.25)	
Thoracic	379 (12.70)	35 (8.37)	344 (13.41)	
Hepatobiliary surgery	217 (7.27)	26 (6.22)	191 (7.44)	
Orthopedic	326 (10.92)	43 (10.28)	283 (11.03)	
cerebral surgery	119 (3.99)	30 (7.18)	89 (3.47)	
Urology	988 (33.11)	128 (30.62)	860 (33.52)	
Other	413 (13.84)	31 (7.41)	382 (14.89)	0.000
Laboratory values				
Hemoglobin, g/dl	121.30 ± 18.97	122.88 (17.63)	121.04 (19.17)	0.193
Lymphocyte, %	0.19 ± 0.10	0.19 (0.10)	0.19 (0.09)	0.541
APTT	36.62 ± 6.31	36.42 (5.69)	36.66 (6.41)	0.616
D-dimer, mg/ml	2.31 ± 2.56	2.33 (2.48)	2.31 (2.58)	0.903
C-reactive protein, mg/l	3.39 (4.76)	3.37 (4.99)	3.40 (4.72)	0.942
Creatine kinase, U/l	87.1 (121.74)	76.23 (87.05)	88.9 (126.45)	0.175
Bilirubin	17.92 ± 2.87	18.12 (3.47)	17.89 (1.54)	0.953
CK-MB	20.83 ± 6.24	26.45 ± 7.39	19.5 ± 4.5	0.019
Myoglobin	80.26 ± 31.21	203.67 ± 34.39	84.65 ± 20.47	0.027
Lactate dehydrogenase, U/l	2.38 ± 0.81	2.35 (0.82)	2.40 (0.80)	0.924
Creatinine, mg/dl	80.81 ± 33.88	77.86 ± 32.91	81.29 ± 34.02	0.765
Albumin, g/dl	33.9 ± 4.80	32.93 (5.19)	34.90 (4.74)	0.698
Sodium, mEq/l	139.15 ± 3.89	139.47 ± 3.57	139.10 ± 3.94	0.919
BNP	343.21 ± 28.7	647.96 (18.01)	321.28 (34.78)	0.043
Tachycardia (heart rate >100 beats/min)	1,012 (33.91)	218 (52.15)	794 (30.94)	0.261
Hypotension (MAP <65 mm Hg)	538 (18.03)	124 (29.66)	414 (16.13)	0.000
SBP >160 mm Hg	946 (31.70)	184 (44.02)	762 (29.70)	0.000

*ACEI, angiotensin converting enzyme inhibitor; ARB, angiotensin receptor blocker; ASA, American Society of Anesthesiologists; BNP, COPD, chronic obstructive pulmonary disease; MAP, mean artery pressure; METS, metabolic Equivalent of Task; RCRI revised cardiac risk index; SBP, systolic blood pressure*.

### Myocardial Injury and Clinical Outcomes After Surgery

The overall incidence of PMI was 418/2,984 (14%). In clinical presentation, the ECG changes within 7 days in patients experiencing a PMI were shown in Table 2. Among the surgical patients who suffered PMI, 156 (37.32%) patients had palpitations and forty (9.57%) patients had clinical ischemic symptoms. The most common ischemic ECG findings were T wave inversion (32.54%) and ST-segment depression (24.4%). Twenty patients were with ECG findings of LBBB and the mortality at 30 days was as high as 10%. In multivariable analyses, coronary artery disease [adjusted OR (aOR) 1.92 95% CI (1.43–2.69)], chronic heart failure [aOR 2.16 95% CI (1.48–4.15), and hypotension [aOR 2.79 95% CI (1.88–4.20) were independently associated with the development of PMI (Table 3).

**Table 2:** Clinical presentation, ECG changes within 7 days in patients experiencing a postoperative myocardial injury (PMI).

	**Prevalence *n* (%)**	**Mortality at 30 days *n* (%)**
Ischemic symptoms	40(9.57)	0
Dyspnea	34(8.13)	0
Typical chest pain	18(4.31)	1(5.5)
Atypical, but still possible ischemic symptoms	78(18.66)	0
Palpitations	156(37.32)	0
Nausea and vomiting	44(10.53)	0
Edema	30(7.18)	0
New ECG findings		
ST-segment elevation	40(9.57)	3(7.5)
ST-segment depression	102(24.40)	0
T-wave inversion	136(32.54)	0
New pathological Q waves	28(6.70)	0
LBBB	20(4.78)	2(10)
Any ischemic ECG changes	320(76.56)	0
PMI fulfilling additional criteria for spontaneous AMI	48(11.48)	2(8.33)

*AMI, acute myocardial infarction; LBBB, left bundle branch block*.

**Table 3:** Multivariable predictors of myocardial injury after non-cardiac surgery.

**Variable**	**Adjusted OR (95% CI)**
Coronary artery disease	1.92 (1.43–2.69)
Hypertension	1.06 (0.77–1.45)
Diabetes Mellitus	1.16 (0.82–1.63)
COPD	1.04 (0.89–1.50)
Cancer	0.65 (0.41–1.09)
Hypotension	2.79 (1.88–4.20)
Chronic heart failure	2.16 (1.48–4.15)
Atrial fibrillation	0.70 (0.35–1.41)
renal insufficiency	2.05(1.60–3.78)

*COPD, chronic obstructive pulmonary disease; OR, odds ratio*.

**Figure 1:**
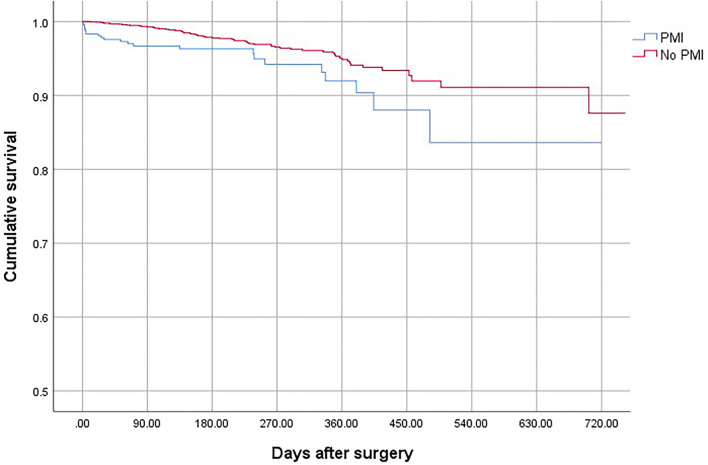
The overall mortality of the patients with and without PMI.

**Figure 2:**
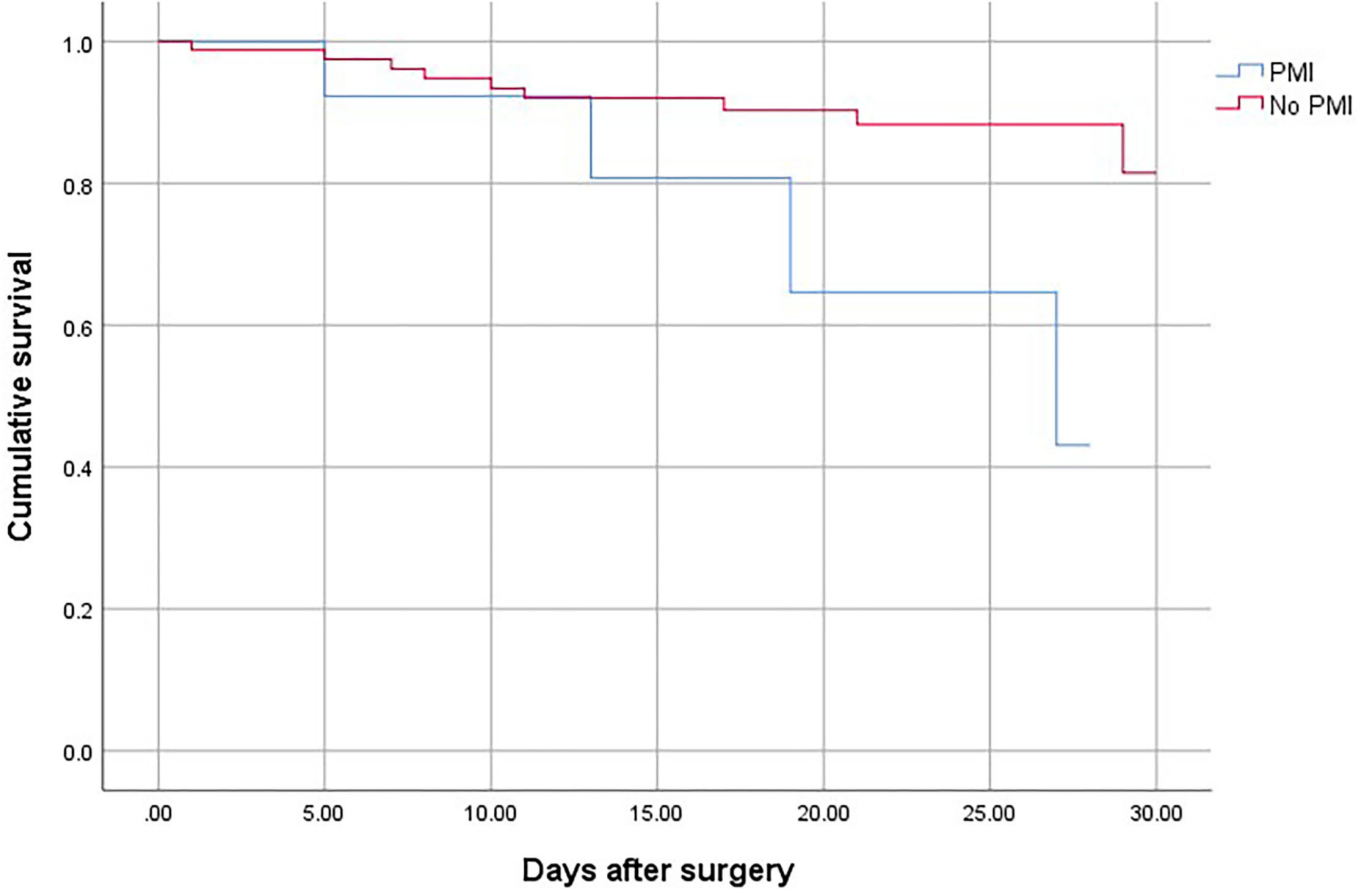
The 30-day mortality for surgical patients with and without PMI.

**Figure 3:**
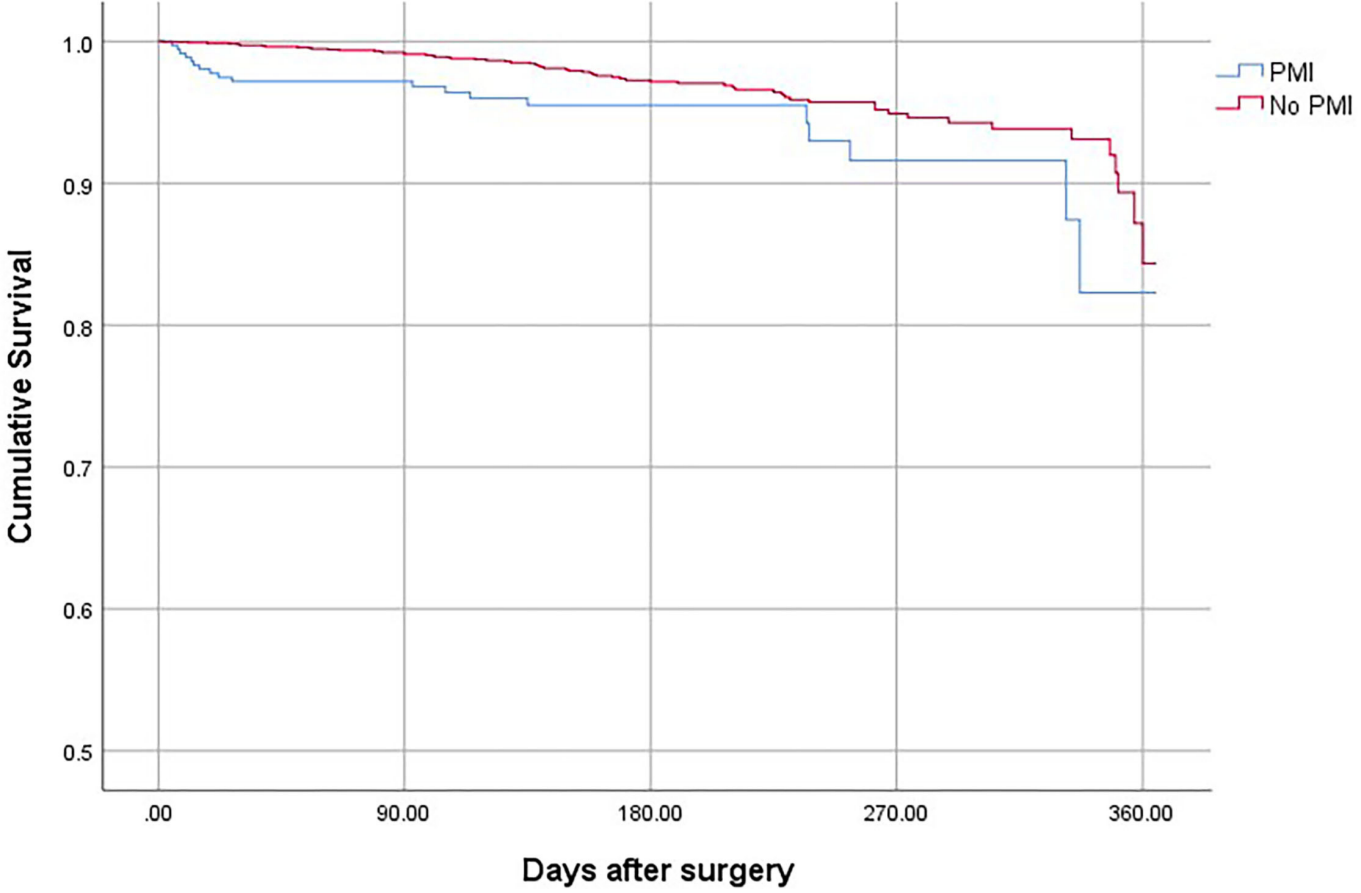
The 1-year mortality for surgical patients with and without PMI.

Patients who suffered from PMI were at higher risk of death (OR, 2.69; 95% CI, 1.78–3.65; *P* < 0.001), non-fatal cardiac arrest (OR,4.52; 95% CI, 2.63–7.64; *P* < 0.001), congestive heart failure (OR, 10.25; 95% CI, 7.86–13.40; *P* < 0.001), and stroke (OR, 3.06; 95% CI, 2.62–4.31; *P* < 0.001) compared with patients who did not develop PMI (Table 4). The overall mortality was 3.15% (Figure 1). The 30-day and 1-year mortality for surgical patients with and without PMI was shown in Figures 2, 3. The 30-day (0.96 vs.0.35%; OR 3.46; 95% CI, 1.49–7.98; *P* < 0.001) and 1-year mortalities (5.37 vs. 2.60%; OR 2.35; 95% CI, 1.12–6.53; *P* < 0.001) were significantly higher in patients who developed PMI compared with those who did not develop PMI.

**Table 4:** Thirty-day outcomes.

**Outcomes**	**No PMI (*n* = 2,566, %)**	**PMI (*n* = 418, %)**	**Unadjusted OR (95% CI)**
Mortality	9 (0.35)	4 (0.96)	2.69 (1.78–3.65)
Nonfatal cardiac arrest	8 (0.31)	8 (1.9)	4.52 (2.63–7.64)
Congestive heart failure	52 (2.03)	42 (10.05)	10.25 (7.86–13.40)
Stroke	31 (1.2)	16 (3.83)	3.06 (2.62–4.31)
Composite of major events	90 (3.50)	61 (14.59)	4.15 (2.76–7.56)

*OR, odds ratio*.

### Perioperative Medical Therapy

Patients were prescribed with low molecular heparin in 18.13% of cases, antiplatelet therapy in 30.7% of cases, oral anticoagulation in 9.42%, an angiotensin-converting enzyme (ACE) inhibitor in 19.7%, a statin in 34.75%, and a beta-blocker in 11.16%. As shown in Table 5, patients who developed PMI were more likely to have received low molecular heparin, anti-plantlet therapy, beta-blocker, early coronary angiography, and statin than patients who did not develop PMI. There were no differences in the prescribing frequency of Angiotensin-converting-enzyme inhibitors (ACEI)/ angiotensin II receptor blockers (ARB) , oral anticoagulation. Overall, low molecular heparin was prescribed in 31.05% (130/418) of PMI cases. Dalteparin sodium 2,500 IU was injected subcutaneously every 12 h. The incidence of major bleeding was 15.38% (20/130) in the entire group. However, four (3.07%, 4/130) patients, who received low molecular heparin therapy, died of massive hemorrhage of the gastrointestinal tract, all of them suffered from acute myocardial infarction.

**Table 5:** Perioperative medical therapy in patients with and without PIM.

	**All patients (*N* = 2,984, %)**	**PMI (*n* = 418, %)**	**No PMI (*n* = 2,566, %)**
Low molecular heparin	541 (18.13)	130 (31.05)	411 (16.02)
Anti-platelet therapy	916 (30.70)	326(y77.99)	590 (22.99)
beta-blocker	333 (11.16)	192 (45.93)	141 (5.49)
ACEI/ARB	588 (19.7)	75 (17.94)	513 (19.99)
Oral Anticoagulation	281 (9.42)	50 (11.96)	231 (9)
Early coronary angiography	34 (1.14)	21 (5.02)	13 (0.55 )
Statin	1,037 (34.75)	293 (70.10)	744 (28.99)
DAPT	270 (9.05)	142 (33.97)	128 (4.98)
Combinations
No Aspirin or Statin	378 (12.67)	121 (28.94)	257 (10.01)
APT + Statin	677 (22.69)	292 (69.85)	385 (15.00)
DAPT + Statin	103 (3.45)	26 (6.22)	77 (3.00)

*APT, anti-platelet therapy; DAPT, dual anti-platelet therapy; ARB, angiotensin receptor blocker; ACEI, angiotensin-converting enzyme inhibitors*.

## Discussion

### Principal Findings

In this retrospective study of 2,984 patients 80 years of age or older undergoing non-cardiac surgery, the incidence of PMI was 14%, which was higher than that seen in a large national database (8%) (3). The 30-day mortality was 0.43%, which was much lower than that seen in other studies (11, 12). The observed 30-day mortality after surgery ranges between 1 and 10% (3, 12–17).

Approximately 50% of PMI occurs on the day of surgery, and 18% on the second postoperative day. Only 22% of patients with PMI fulfilled the universal definition of myocardial infarction. A minority (9.57%) of patients suffering PMI experienced an ischemic feature. Any ischemic ECG changes were presented in 76.56% of patients with PMI. Therefore, nearly 90% of PMI would, probably, have gone undetected without systematic troponin and ECG monitoring after surgery. Patients with PMI had a greater burden of cardiovascular events than patients without PMI. In comprehensive elevations of cardiac troponin in combination with ischemic symptoms, ECG changes are helpful to identify PMI.

### Perioperative Myocardial Infarction/Injury

The combination of aging and multi-comorbidities, such as cardiovascular disorders or renal insufficiency, is the principal factor increasing surgical stress in older patients. The elderly patient who undergoes surgery has decreased ability to compensate and maintain homeostasis when the body is stressed. The perioperative management of old patients with multiple conditions is a great challenge for hospital physicians.

The perioperative risk of patients is markedly increased due to the cardiac condition itself and co-morbidities. In the present study, multivariable analyses showed that coronary artery disease, chronic heart failure, and hypotension were independently associated with the development of PMI. Co-management model by surgeons and geriatricians is helpful with perioperative management of cardiovascular complications and has the potential to improve outcomes. In our study, the incidence of PMI was higher and the 30-day mortality was lower than that seen in other studies (11, 12). A system review showed orthogeriatric collaboration to improve mortality after hip repair (18). One recent meta-analysis demonstrated that orthogeriatric care models reduced length of stay, in-hospital mortality, 1-year mortality, and delirium of patients with hip fracture and may reduce complications and cost (19).

The PMI is a complex syndrome with multiple different underlying etiologies, including type 1 myocardial infarction (T1MI), and emphasis on the causal relationship of plaque disruption with coronary atherothrombosis; while in type 2 myocardial infarction (T2MI), settings with oxygen demand and supply imbalance are unrelated to acute coronary atherothrombosis (6). The PMIs, after non-cardiac surgery, are largely considered type 2 infarction. The OPTIMUS and Coronary CTA VISION Studies showed that, at most, a quarter to a third of PMIs are due to thrombosis, whereas at least two-thirds to three-quarters are likely due to supply-demand mismatch (20, 21). In the present study, 22% of patients with PMI fulfilled the universal definition of myocardial infarction. A patient with a history of previous coronary artery disease merits special attention as it is a strong predictor of PMI. Given the super old age and higher prevalence of concomitant coronary artery disease in a series of patients with PMI, we suggest consideration of non-invasive evaluation for ischemia if appropriate. The risk and benefit of coronary interventions should be taken into account more cautiously in patients, who are at risk of bleeding shortly after surgery, because withdrawing antiplatelet therapy may lead to in-stent thrombosis (22, 23).

Another issue of perioperative risk is bleeding (24). Of note, 31.05% of patients with PMI received therapy of low molecular weight heparin (LMWH). The median duration of postoperative LMWH administration was 7 days. The incidence of major bleeding was 15.38% and 30-day mortality was 3.07%. Management of anticoagulation during invasive procedures varies widely and remains challenging and controversial. Based on these findings, it was concluded that LMWH should be used to decrease the risk of thromboembolic events without increasing the risk of bleeding. The risks of bleeding for any surgical procedure must be weighed against the benefit of remaining on anticoagulants on a case-by-case basis.

The optimal management of patients with PMI remains an area of the ongoing investigation. A multidisciplinary approach and expert guidance on the assessment and medical management of multi-morbidity, pump flow control, hemodynamic monitoring, anticoagulation strategies, infection, and bleeding prevention strategies are considered important. Appropriate use of intravenous fluids to maintain fluid and electrolyte balance is important for normal organ function after surgery. Optimal perioperative medical therapy should be adopted for the prevention and management of PMI, including antiplatelet agents, β-Blocker (25–29), statins (12, 30–34), ACE inhibitors, and anticoagulants. These patients can benefit from intensification of assessment and medical management of multi-morbidity during the perioperative period (35).

### Strengths and Weaknesses of Our Study

The main strength of the present study is that it includes a large sample of surgical patients aged over 80 years, which allowed us to demonstrate the incidence and prognosis of the PMI diagnosis in elderly surgical patients, to show the advantage of the co-management model by geriatricians and surgeons in mortality. There are several limitations of this study. First, this study was a retrospective study, excluding patients without troponin T measurement, thus, leading to a remarkable risk of selection bias. Second, the effects of troponin levels on outcomes were not analyzed. Third, the subjects were from a single health center and most of them were male.

## Conclusion

The incidence of PMI in the very old surgical patients was relatively high. The PMI is associated with an increased risk of 30-day and 1-year mortality. The treatment strategy is firstly focused on the risk factors and guideline-directed medical therapy. Cardiovascular medical treatments, such as antiplatelets, β-blockers, statins, and angiotensin-converting enzyme inhibitors, have shown improved outcomes in patients with PMI. The surgical risk of bleeding should be considered before using antiplatelets and anticoagulation.

## Data Availability Statement

The original contributions presented in the study are included in the article/supplementary material, further inquiries can be directed to the corresponding authors.

## Ethics Statement

The studies involving human participants were reviewed and approved by the Ethics Committee of Chinese PLA General Hospital. The patients/participants provided their written informed consent to participate in this study.

## Author Contributions

LG, LC, and BW: conceptualization and methodology. LG, JH, and LC: investigation and writing—original draft. LG, LC, and BW: writing—review and English editing. LG, RW, and RC: supervision. All authors contributed to the article and approved the submitted version.

## Funding

This work was supported by the Military Healthcare Fund 19BJZ35.

## Conflict of Interest

The authors declare that the research was conducted in the absence of any commercial or financial relationships that could be construed as a potential conflict of interest.

## Publisher's Note

All claims expressed in this article are solely those of the authors and do not necessarily represent those of their affiliated organizations, or those of the publisher, the editors and the reviewers. Any product that may be evaluated in this article, or claim that may be made by its manufacturer, is not guaranteed or endorsed by the publisher.
